# Dual-shot dynamics and ultimate frequency of all-optical magnetic recording on GdFeCo

**DOI:** 10.1038/s41377-020-00451-z

**Published:** 2021-01-06

**Authors:** Sicong Wang, Chen Wei, Yuanhua Feng, Hongkun Cao, Wenzhe Li, Yaoyu Cao, Bai-Ou Guan, Arata Tsukamoto, Andrei Kirilyuk, Alexey V. Kimel, Xiangping Li

**Affiliations:** 1 Guangdong Provincial Key Laboratory of Optical Fiber Sensing and Communications, Institute of Photonics Technology, Jinan University, Guangzhou, 510632 China; 2 Institute for Molecules and Materials, Radboud University, Heyendaalseweg 135, 6525 AJ Nijmegen, The Netherlands; 3 Department of Electronic Engineering, College of Information Science and Technology, Jinan University, Guangzhou, 510632 China; 4 Institute of New Energy Technology, Department of Electronic Engineering, College of Information Science and Technology, Jinan University, Guangzhou, 510632 China; 5 College of Science and Technology, Nihon University, 7-24-1 Funabashi, Chiba, 274-8501 Japan; 6 FELIX Laboratory, Radboud University, Toernooiveld 7, 6525 ED Nijmegen, The Netherlands

**Keywords:** Magneto-optics, Optical data storage

## Abstract

Although photonics presents the fastest and most energy-efficient method of data transfer, magnetism still offers the cheapest and most natural way to store data. The ultrafast and energy-efficient optical control of magnetism is presently a missing technological link that prevents us from reaching the next evolution in information processing. The discovery of all-optical magnetization reversal in GdFeCo with the help of 100 fs laser pulses has further aroused intense interest in this compelling problem. Although the applicability of this approach to high-speed data processing depends vitally on the maximum repetition rate of the switching, the latter remains virtually unknown. Here we experimentally unveil the ultimate frequency of repetitive all-optical magnetization reversal through time-resolved studies of the dual-shot magnetization dynamics in Gd_27_Fe_63.87_Co_9.13_. Varying the intensities of the shots and the shot-to-shot separation, we reveal the conditions for ultrafast writing and the fastest possible restoration of magnetic bits. It is shown that although magnetic writing launched by the first shot is completed after 100 ps, a reliable rewriting of the bit by the second shot requires separating the shots by at least 300 ps. Using two shots partially overlapping in space and minimally separated by 300 ps, we demonstrate an approach for GHz magnetic writing that can be scaled down to sizes below the diffraction limit.

## Optical magnetization: extreme limits for magnetic data recording with light

The potential for combining the speed of photonics with the advantages of magnetic data storage, using pulsed laser light to control the magnetization of alloys of gadolinium, iron and cobalt (GdFeCo), has been explored and quantified. Achieving ultrafast and energy-efficient optical control of magnetism could revolutionize information-processing technology. Researchers led by Xiangping Li at Jinan University in China and Alexey V. Kimel at Radboud University in The Netherlands have experimentally determined the fastest possible rate of the optical reversal of magnetization achieved by this promising new technology. They suggest data switching repetition rates of up to three billion cycles per second (3 GHz) are feasible. They also propose a method to achieve spatial resolution of data recording at scales below light’s ‘diffraction limit’, which is generally believed to restrict the attainable resolution.

## Introduction

The development of ultrafast all-optical switches has long been a popular topic in photonics^1,2^, whereas the speed of magnetization reversal triggered by means other than magnetic fields has recently attracted intense interest in spintronics^3–11^. The discovery of all-optical helicity-dependent switching in metallic GdFeCo has promised a merger of the fields of photonics and spintronics, paving the way for faster and more energy-efficient information-processing technologies^12,13^. However, the real potential of all-optical switching is still poorly understood, because it is still unclear whether magnetic switching by light can keep up with the GHz frequencies required by photonics technologies^14–19^. Another serious obstacle is the scepticism regarding the scalability of all-optical magnetic switching down to the sizes of spintronic devices, which are well below the diffraction limit^19–24^. In this study, employing dual laser pulses and time-resolved optical imaging, we reveal the maximum repetition rate of all-optical magnetic switching in Gd_27_Fe_63.87_Co_9.13_ and propose an approach for ultrafast all-optical writing scalable below the diffraction limit.

## Results

### The final magnetization states of Gd_27_Fe_63.87_Co_9.13_ under dual-shot excitation

**Fig. 1:**
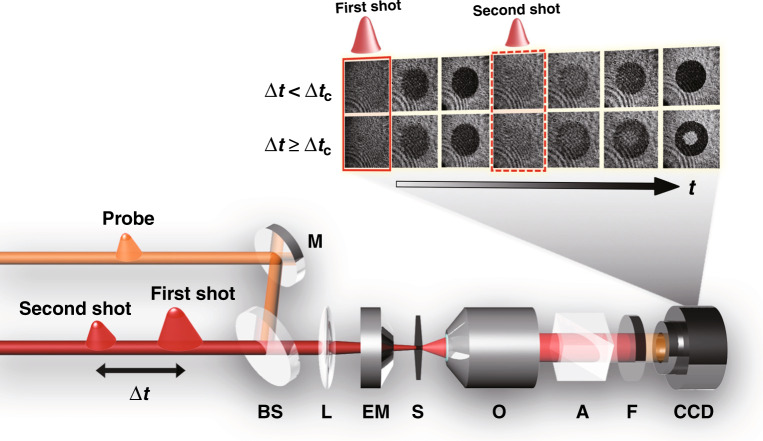
Schematic of the dual-shot magnetization dynamics acquired by a time-resolved magneto-optical imaging system. M: mirror, BS: beam splitter, L: focal lens, EM: electromagnet, S: sample, O: objective, A: analyser, F: colour filter, CCD: charge-coupled device. The inset illustrates the magnetization dynamics of Gd_27_Fe_63.87_Co_9.13_ under dual-shot excitation with certain conditions. ∆*t* is the shot-to-shot separation between the first and second pulses. ∆*t*_c_ is the critical time delay. Rewriting or restoration of the switched magnetization may occur in no less than this amount of time. The solid and dashed red frames indicate the images acquired at the arrivals of the first and second shots, respectively

A time-resolved magneto-optical imaging system for studying dual-shot magnetization dynamics is shown in Fig. 1. Linearly polarized dual-pump (800 nm) and probe (650 nm) beams with a pulse width of 40 fs are incident on the sample collinearly through the same focal lens with a low numerical aperture (NA). The lens condenses the pump beams into focal spots with a lateral size down to approximately 160 μm. The separation (∆*t*) between two consecutive pump pulses varies with picosecond resolution. The probe beam is employed to record the magnetization states at different time delays with respect to the pump pulses using a magneto-optical microscope. This microscope is sensitive to the out-of-plane orientation of the magnetization of the FeCo sublattice through the magneto-optical Faraday effect^25^. Magnetic domains with magnetization parallel (‘up’) or antiparallel (‘down’) to the sample normal are shown as white or black regions, respectively. The initial magnetization state is white, as shown in the inset of Fig. 1. The images acquired at the arrival of the first and second shots are indicated by the solid and dashed red frames, respectively. The experiments were performed at room temperature in air. Detailed illustrations of the experimental setup are shown in the Methods section and in Supplementary Fig. S1.

**Fig. 2:**
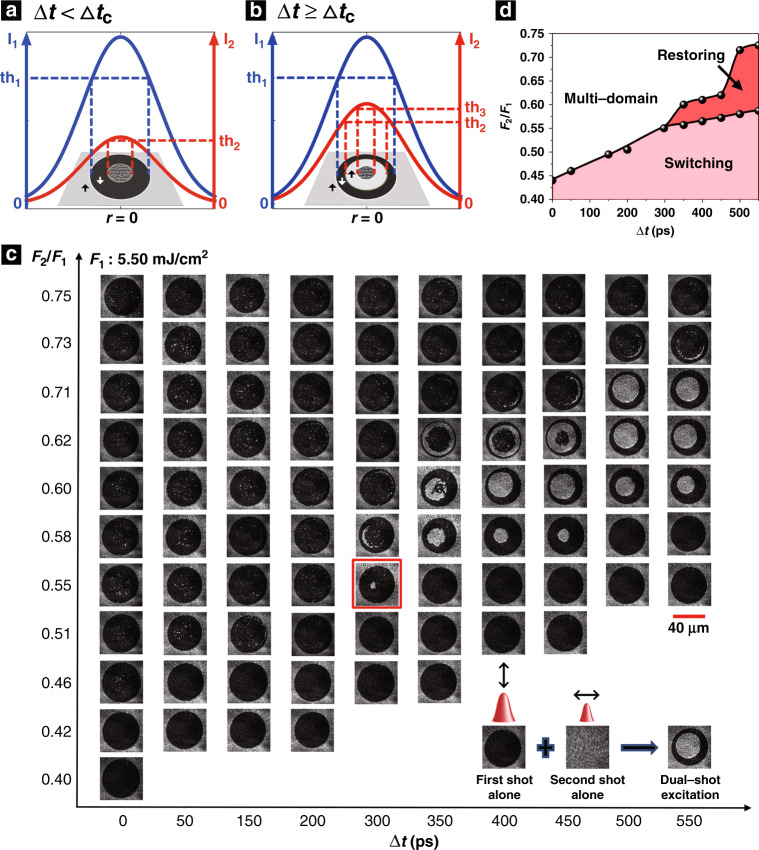
The final magnetization states of Gd_27_Fe_63.87_Co_9.13_ under dual-shot excitation. **a**, **b** Sketches of the final magnetization states of Gd_27_Fe_63.87_Co_9.13_ under dual-shot excitation when ∆*t* < ∆*t*_c_ (**a**) and when ∆*t* ≥ ∆*t*_c_ (**b**). **c** The final state distributions of Gd_27_Fe_63.87_Co_9.13_ under dual-shot excitation versus ∆*t* and the pulse fluence ratio (*F*_2_/*F*_1_) between the two pulses. The red frame indicates the onset of rewriting a magnetic bit by dual-shot excitation with minimal pulse-to-pulse separation. The scale bar is 40 μm. **d** The corresponding phase diagram

First, we studied the final magnetization states induced by various dual shots that were acquired a few seconds after excitation. Sketches of the final magnetization states of Gd_27_Fe_63.87_Co_9.13_ under dual-shot excitation in different conditions are shown in Fig. 2a, b. The blue and red curves illustrate the Gaussian spatial profiles of the light intensities of the first and second shots, respectively. The black and white arrows denote the magnetization orientations in the corresponding regions. th_1_, th_2_, and th_3_ are the intensity thresholds above which a different magnetization state may appear with respect to its peripheral region. Notably, the intensity thresholds for inducing magnetic switching and multi-domain states by the second shot are markedly lower than those of the first shot alone, as illustrated in Fig. 2b.

Figure 2c shows the dependence of the final magnetization state of Gd_27_Fe_63.87_Co_9.13_ under dual-shot excitation on the shot-to-shot separation (∆*t*) and the ratio between the average pulse fluences of the two pump pulses (*F*_2_/*F*_1_). The average pulse fluence is defined as the pulse energy divided by the beam area on the sample. The black arrows in the inset indicate the polarizations of the two pump pulses. The polarizations are set to be orthogonal to avoid any interference between the pulses. *F*_1_ is fixed at approximately 5.50 mJ/cm^2^, at which the first shot alone is able to perform all-optical switching to a black state (see the inset in Fig. 2c). To demonstrate the effect of the second pulse, we take *F*_2_/*F*_1_ = 0.62 and Δ*t* = 500 ps as an example. The second pulse, with a fluence of *F*_2_/*F*_1_ = 0.62, is not intense enough to induce magnetization switching on its own. Surprisingly, a black annular pattern of magnetization is formed by dual-shot excitation with a shot-to-shot separation of 500 ps. The white region in the centre indicates that the magnetization that was already switched to the black state by the first shot in the centre can be restored to the white state by the second shot even though the fluence of the latter is below the single-shot switching threshold. When ∆*t* < 300 ps, the dual-shot pulses either switch the magnetization to the black state or demagnetize the sample. Only when the second shot impinges on the sample 300 ps after the first shot or later does the white area in the centre begin to reappear. This shot-to-shot separation is defined as the critical time delay (∆*t*_c_) required for rewriting a magnetic bit. Similar results are also observed when the polarizations of the dual pulses are parallel (see Supplementary Fig. S2). The multi-domain state shown in Fig. 2c looks similar to a switched state with some smaller regions in the white state (see Supplementary Note 1 and Supplementary Fig. S3), which is probably because of the specific composition and the production processes of the sample.

The corresponding phase diagram of the final magnetization state obtained by these time-delayed dual pulses is shown in Fig. 2d. Rewriting or restoring to the initial magnetization state occurs only in a narrow window where the fluence of the second pulse is lower than that of the first pulse and the pulse-to-pulse separation is no less than Δ*t*_c_ ≈ 300 ps. With a longer ∆*t*, the values of the fluence ratio (*F*_2_/*F*_1_) for rewriting increase and the corresponding fluence range expands. To explain these findings, we note that the efficiency of all-optical switching depends on the sample temperature. In particular, it has been shown that switching in GdFeCo ferrimagnetic alloys is the most efficient in the vicinity of the magnetization compensation point (*T*_M_)^5,26,27^. We have experimentally shown that the maximum temperature at which all-optical switching can be observed is ~470 K (see Supplementary Note 2 and Supplementary Fig. S4). Therefore, if the first pump pulse increases the sample temperature above 470 K, the second pump pulse will be able to switch the magnetization only after some time required to cool the sample down to 470 K. Hence, the pulse-to-pulse separation Δ*t*_c_ ≈ 300 ps required for reversible switching can be related to the cooling-down time. This conclusion is further supported by modelling the dynamics of the electron and lattice temperatures launched by dual-shot excitation (see Supplementary Note 3 and Supplementary Fig. S5). As the range of the fluence of the first pump *F*_1_ required for the switching is relatively narrow, the critical time delay Δ*t*_c_ obtained within the investigated pump fluence window seems to be independent of *F*_1_ and remains constant at ~300 ps, as shown in Supplementary Fig. S6. This observation shows that, at least for the studied sample, the repetition rate of all-optical magnetic switching can be increased up to 3 GHz. A significant decrease in the fluence of the first pump *F*_1_ by optimizing the sample composition^12,13^ and improving the efficiency of the heat sink may provide ways to obtain a 10–100 times higher repetition rate of switching^10^.

### The magnetization dynamics of Gd_27_Fe_63.87_Co_9.13_ observed by the time-resolved imaging technique

**Fig. 3:**
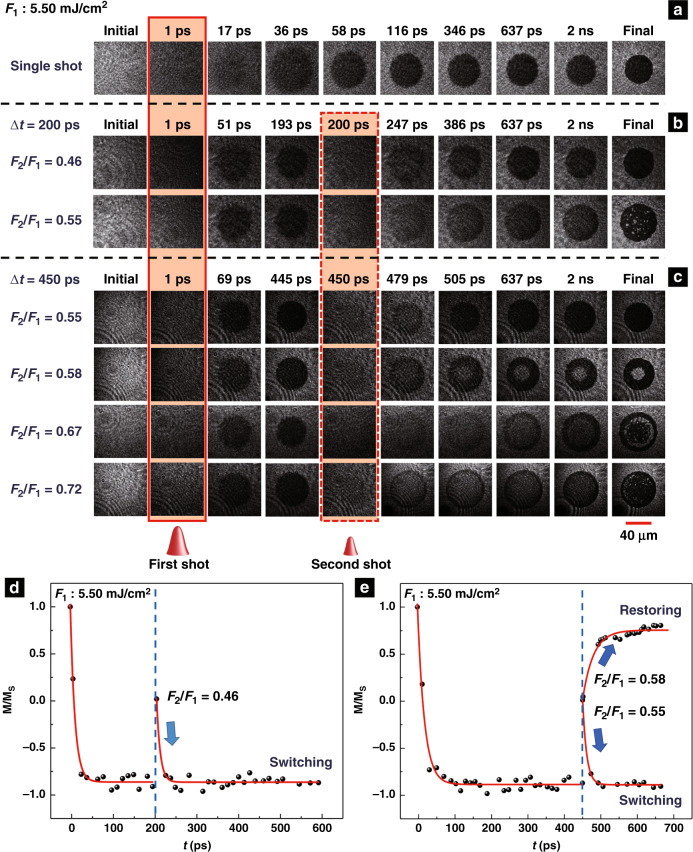
The magnetization dynamics of Gd_27_Fe_63.87_Co_9.13_ observed by the time-resolved imaging technique. **a**–**c** The magnetization dynamics of Gd_27_Fe_63.87_Co_9.13_ under single-shot excitation (**a**) and dual-shot excitation (**b**, **c**). The solid and dashed red frames indicate the images acquired at the arrivals of the first and second shots, respectively. The scale bar is 40 μm. **d**, **e** The normalized magnetization in the centres of the switched areas extracted from the images in **b**, **c**. The red lines are guides for the eye. ∆*t* in **b** and **d**, and in **c** and **e** is 200 ps and 450 ps, respectively

To gain insight into the dual-shot magnetization dynamics in Gd_27_Fe_63.87_Co_9.13_, time-resolved magneto-optical imaging experiments were performed. The magnetization dynamics of Gd_27_Fe_63.87_Co_9.13_ under single-shot excitation and time-delayed dual-shot excitation are shown in Fig. 3a and Fig. 3b–e, respectively. Figure 3d, e depict the normalized magnetization in the centre of the switched regions, as extracted from the images in Fig. 3b, c, respectively. The value of the normalized initial magnetization, shown as the white domain, equals 1. *M*_s_ is the saturated magnetization that can be induced by the external magnetic field. ∆*t* in Fig. 3b, d and in Fig. 3c, e is 200 ps and 450 ps, respectively. Similar to the previous results in ref. ^5^, after single-shot or first-shot excitation, as indicated in the solid red frame, the metastable state corresponding to the reversed magnetization is reached within 60 ps and reliable magnetic writing is perceived as completed after 100 ps, as shown in Fig. 3a, d, e and in Supplementary Movie S1.

For dual-shot excitation, a strongly non-equilibrium state appears again when the second shot impinges on the sample, as indicated in the dashed red frame in Fig. 3b, c. As long as ∆*t* < ∆*t*_c_ (e.g., when ∆*t* = 200 ps), a second shot with a low fluence (*F*_2_/*F*_1_ < 0.51) can trigger only transient demagnetization and the magnetization subsequently relaxes back to the black state. Otherwise, a second shot with a larger fluence (*F*_2_/*F*_1_ ≥ 0.51) can induce multi-domain final states (see Supplementary Movies S2 and S3). From Fig. 3d, it can be seen that when *F*_2_/*F*_1_ = 0.46, after the excitation of the second shot, the metastable state corresponding to the black domain is likewise reached within 60 ps. In contrast, as long as ∆*t* ≥ ∆*t*_c_ (e.g., when ∆*t* = 450 ps), all-optical switching, restoring, and multi-domain final states appear, depending on the fluence ratio, as shown in Fig. 3c. From Fig. 3e, it can be seen that the magnetization enters the switching state and the restoring state when *F*_2_/*F*_1_ = 0.55 and *F*_2_/*F*_1_ = 0.58, respectively (see Supplementary Movies S4 and S5). The second shot induces magnetization dynamics similar to those of the first shot. Similarly, their corresponding metastable states are reached within approximately 60 ps after the arrival of the second shot. Supplementary Movies S6 and S7 record the magnetization dynamics when *F*_2_/*F*_1_ = 0.67 and *F*_2_/*F*_1_ = 0.72, respectively. Additional examples of the magnetization dynamics by dual-shot excitation with different shot-to-shot separations are shown in Fig. S7, which presents similar magnetization evolution processes.

### Proof-of-principle demonstrations of sub-diffraction all-optical switching

**Fig. 4:**
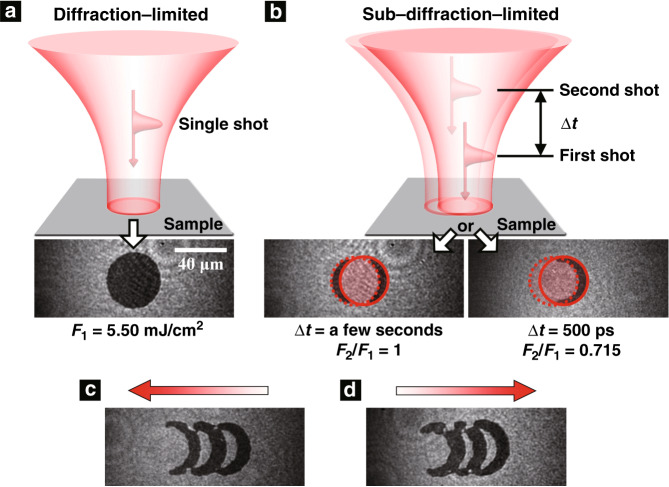
Proof-of-principle demonstrations of sub-diffraction all-optical switching. **a** The final magnetization state induced by a single pulse. **b** The final magnetization states induced by a pair of spatially offset and time-delayed dual pulses with ∆*t* = a few seconds and ∆*t* = 500 ps. The solid and dashed circles indicate the irradiation areas of the first and second pulses, respectively. **c**, **d** The one-dimensional sub-diffraction all-optical switching array recorded through the dual-shot method (∆*t* = 500 ps) by sweeping the dual pulses from the right side to the left side of the sample (**c**) or vice versa (**d**). The red arrows indicate the sweeping directions. The scale bar is 40 μm

Harnessing these ultrafast dual-shot dynamics, we demonstrate a proof-of-principle that would allow sub-nanosecond sub-diffraction magnetic switching with light. Figure 4a shows the final magnetization state induced by a single pump pulse. An intact round black switched region with a lateral size of 40 μm is clearly formed, given the diffraction-limited focusing condition of a low NA focal lens (NA ≈ 0.0025). In contrast, utilizing the dual-shot effect, an area subtraction of the switched magnetization can be reproducibly achieved, and the corresponding lateral size in the horizontal direction can be far below the diffraction limit once the second pulse is spatially offset with respect to the first pulse, as shown in Fig. 4b. Dual-shot excitation with carefully controlled pulse separation (∆*t*) and fluences (*F*_2_/*F*_1_) (e.g., ∆*t* = 500 ps and *F*_2_/*F*_1_ = 0.715) enables the creation of a distinct crescent-shaped magnetic bit with a lateral size of ~8 μm in the horizontal direction where the overlapping region of the two consecutive pulses that was initially switched to the black state by the first shot is restored to the white state by the second shot. In stark contrast, a long-time-delayed successive pump pulse with the same fluence as that of the first shot can achieve a similar area subtraction effect, but a side effect in this case is an annoying crescent-shaped magnetic bit on the complementary site concomitantly owing to the high fluence of the successive pulse (Fig. 4b).

Compared with diffraction-limited all-optical switching by single-shot excitation, the switching size is reduced substantially in the horizontal direction through the dual-shot method. This promises to pave the way towards achieving magnetization control in a super-confined region through light, one of the significant potential applications of which is to perform magnetic recording beyond the diffraction limit. Furthermore, a one-dimensional sub-diffraction all-optical switching array with a bit interval of ~16 μm is recorded through the dual-shot method, as shown in Fig. 4c, d, where the partially overlapping dual pulses sweep the sample from the right side to the left side or vice versa. Even though the focal spot of each pulse is still diffraction-limited, adjacent magnetic bits can be distinctly recorded with sub-diffraction bit intervals without any adverse effects induced by contiguous dual-shot exposure, which has the merit of achieving long-sought high-density magnetic recording by all-optical means.

## Discussion

A higher repetition rate is always expected in realistic applications. Taking into account the fact that the maximum repetition rate of all-optical switching is defined by the cooling time, one may anticipate several possibilities for a 10- to 100-fold increase in the former. In addition to improvements in the heat sink facilitating faster cooling down, it is also possible to decrease the fluence required for the switching by optimizing the composition of the ferrimagnetic medium. However, we cannot ignore the fact that scaling the all-optical switching down to sub-100 nm will surely affect the processes of heat transfer, cooling down, and, consequently, repetitive switching. Understanding submicrometer and nanoscale magnetization dynamics triggered by multiple-shot excitation is the next challenge for theoretical and experimental studies in ultrafast magnetism. On the other hand, applying the dual-shot method to other kinds of magneto-optical materials that possess the potential to realize single-shot switching could be an alternative to further enhance the repetition rate of all-optical switching.

Although we have found that the maximum temperature at which all-optical switching can be observed is ~470 K in Gd_27_Fe_63.87_Co_9.13_, the explicit underlying physics and deterministic theory are still open to the community and will be worthy subjects for future in-depth theoretical studies and experimental validations.

In conclusion, a time-resolved imaging technique reveals the magnetization dynamics triggered in Gd_27_Fe_63.87_Co_9.13_ by dual-shot laser excitation and defines the maximum repetition rate of all-optical magnetic recording on the medium. Our findings demonstrate the potential of all-optical magnetic writing with a repetition rate of up to 3 GHz and a spatial resolution below the diffraction limit, which fills a knowledge gap and completes missing technology to promote its widespread applicability in the next revolution of information processing. The advanced features observed in this work may favour the realization of spatially and temporally confined magnetization control through light and greatly promote the development of ultrafast and highly compact devices at the intersection of photonics and spintronics.

## Materials and methods

### Sample preparation

The amorphous ferrimagnetic Gd_27_Fe_63.87_Co_9.13_ sample is grown by magnetron sputtering in a multilayer structure: glass/AlTi (10 nm)/SiN (5 nm)/GdFeCo (20 nm)/SiN (60 nm), where the AlTi layer serves as a heat sink and SiN is used as a buffer and capping layer^12,24^. Thin films of this alloy usually exhibit strong perpendicular magnetic anisotropy and have a Curie point *T*_C_ ≈ 550 K and a magnetization compensation point *T*_M_ ≈ 485 K (see Supplementary Note 2 and Supplementary Fig. S4). A small amount of Co (9.13%) is added to control the perpendicular anisotropy of the film.

### Experimental setup and characterization

A more detailed schematic of the experimental setup is shown in Supplementary Fig. S1. The linearly polarized pump (800 nm) and probe (650 nm) beams come from an amplified Ti:sapphire laser and an optical parametric amplifier, respectively. The repetition rate and the pulse width of the pulses from these two laser sources are 1 kHz and 40 fs, respectively. A pulse picker is used collaboratively to reduce the repetition rate of the pump pulses. Two optical delay lines (ODLs) are introduced to control the optical paths of the probe beam (with a longer ODL) and the second pump beam (with a shorter ODL), and hence control the time delay between the pump–probe pulses (*τ* shown in Fig. S1b) and that between the two pump pulses (∆*t*), respectively. The pump and probe beams are incident on the sample colinearly through the same focal lens, with a low NA of ~0.0025, which condenses the pump beams down to ~160 μm in lateral size and thereby allows the dual-shot magnetization dynamics of Gd_27_Fe_63.87_Co_9.13_ to be measured by finely adjusting the positions of the two ODLs. An electromagnet is used to initialize the magnetization state of the sample. A combination of an objective (×10, NA = 0.25), an analyser, a colour filter that can filter out the pump beams, and a charge-coupled device camera (CoolSNAP MYO) is used to image the magnetic domains of the sample through the magneto-optical Faraday effect.

## Supplementary information

Supplementary

Movie S1

Movie S2

Movie S3

Movie S4

Movie S5

Movie S6

Movie S7
