# Supplementary material for: Dual-shot dynamics and ultimate frequency of all-optical magnetic recording on GdFeCo
Source: Light Sci Appl. 2021 Jan 6;10:8. doi: 10.1038/s41377-020-00451-z (PMC7788082; doi:10.1038/s41377-020-00451-z)
Supplement: Supplementary file 1 — Supplementary [file 41377_2020_451_MOESM1_ESM.docx]

**Supplementary information**

**Dual-shot dynamics and ultimate frequency of all-optical magnetic recording on GdFeCo**

**Sicong Wang^1,2^, Chen Wei^1^, Yuanhua Feng^3^, Hongkun Cao^1^, Wenzhe Li^4^, Yaoyu Cao^1^, Bai-Ou Guan^1^, Arata Tsukamoto^5^, Andrei Kirilyuk^2,6^, Alexey V. Kimel^2*^, and Xiangping Li^1*^**

^1^Guangdong Provincial Key Laboratory of Optical Fiber Sensing and Communications, Institute of Photonics Technology, Jinan University, Guangzhou 510632, China

^2^Radboud University, Institute for Molecules and Materials, Heyendaalseweg 135, 6525 AJ Nijmegen, The Netherlands

^3^Department of Electronic Engineering, College of Information Science and Technology, Jinan University, Guangzhou 510632, China

^4^Institute of New Energy Technology, Department of Electronic Engineering, College of Information Science and Technology, Jinan University, Guangzhou 510632, China

^5^College of Science and Technology, Nihon University, 7-24-1 Funabashi, Chiba 274-8501, Japan

^6^FELIX Laboratory, Radboud University, Toernooiveld 7, 6525 ED Nijmegen, The Netherlands

* Email: Aleksei.Kimel@ru.nl; [xiangpingli@jnu.edu.cn](mailto:xiangpingli@jnu.edu.cn)

**Supplementary notes**

**Note 1**

To characterize the magnetization states shown in the manuscript, the cross-sections of the magnetization are shown in Fig. S3. The multi-domain state looks like a switched state (black state) with some smaller regions in the white state, which means that there probably still remains a significant magnetization state in these spot regions. Nevertheless, compared to the switching state, this state is still less uniformly magnetized, and its formation requires more pulse energy. Additionally, the small white dots are randomly distributed in the region surrounded by the reversed magnetization, which is consistent with the multi-domain states shown in previous studies on GdFeCo^1,2^. This “abnormal” multi-domain state probably comes from the specific composition and the production processes of the sample.

**Note 2**

We discover that a reliable rewriting of a bit by the time-delayed second shot requires to separate the shots by at least 300 ps. Although at this stage we are keeping minds open for explicit underlying physics and deterministic theory, the experimental results outline a complete diagram at which the rewriting condition occurs. In Fig. 2, it clearly unveils that the arrival of the second pulse has to be later than 300 ps after the advent of the first shot.

In order to understand the origin of the findings, we have measured the magnetization compensation temperature (T_M_) of our sample and how the minimum fluence required for single-shot all-optical switching changes as a function of temperature. Fig. S4a shows the normalized magnetization of the FeCo sublattice at different temperatures with an out-of-plane external magnetic field around 800 Oe. It can be seen that a clear domain wall emerges at around 485 K. Moreover, the static hysteresis loop changes its sign as the temperature crosses the point of 485 K, which indicates this is the magnetization compensation temperature of the sample (T_M_ ≈ 485 K).

Fig. S4b shows the dependence of the minimum fluence for single-shot all-optical switching on the sample temperature. It can be seen that when the temperature is below a critical temperature which is about 470 K (T_Critical_), the minimum fluence for single-shot switching decreases linearly. Above T_Critical_, no all-optical switching can be observed even if the pump fluence is increased.

These results provide a clue to explain the observations of ∆t_c_ and the different fluence thresholds of the dual shots to some extent. The all-optical switching in the GdFeCo system is largely perceived as the thermally driven magnetization dynamics by the optical stimulus^3^. As shown in Fig. S4b, the all-optical switching occurs only when the initial temperature is below 470 K. This may indicate why a reliable rewriting of a bit by the time-delayed second shot requires to separate the shots by at least 300 ps. The transient temperature of the sample is very likely to be around 470 K at ∆t_c_ after the excitation of the first shot. Additionally, Fig. S4b shows that the fluence for all-optical switching decreases with higher initial temperature. Therefore, a lower fluence threshold is needed to realize rewriting. As T_M_ depends on the material composition very sensitively, figuring out the distinct relationship between ∆t_c_ and T_M_ by changing the material composition is necessary, which is subject to further investigation.

**Note 3**

We calculated the time evolutions of the electron and lattice temperatures (T_e_ and T_l_) under dual-shot excitation with ∆t = 300 ps, 400 ps, and 500 ps, respectively, based on the two-temperature model which can be expressed as

(S1)

(S2)

where

(S3)

C_e_ = γT_e_ with γ = 700 J/(K^2^m^3^) and C_l_ = 3×10^6^ J/(Km^3^) are the electron and the lattice specific heats, respectively, and G_el-ph_ = 1.7×10^18^ J/(sKm^3^) is the electron-phonon coupling constant^4^. The time constant τ_th_ describes the relaxation back to the initial temperature T_0_ and depends on the interfacial thermal conductance at the contact between the GdFeCo thin film and its adjacent layers. The interfacial thermal conductance between these layers is estimated to be around 10^8^ J/(sKm^2^)^5,6^, and hence τ_th_ is inferred to be around 1 ns^7^, which is generally consistent with the descriptions for metal in Ref. [8,9]. The source term P(t) describes dual-shot excitation with a pulse width of τ = 40 fs. Q(t) indicates the corresponding absorbed energy by the material, where α ≈ 0.5 is the absorption coefficient^2^. Hereby, we only consider the temperature evolutions at the centre of the switched region and hence Eq. (S3) is independent of the space coordinates.

From Fig. S5, it can be seen that after the excitation of the second pump pulse, which can induce the rewriting of the magnetization, the temperature at 2∆t is higher than that at ∆t. This may indicate that the time delay between the second and third pulses for rewriting might be longer than ∆t and hence decrease the actual repetition rate. Nevertheless, these relatively rough calculations are based on an intuitive understanding of the influence of the temperature relaxation on an unoptimized sample with a relatively long cooling time of close to 1 ns.

**Supplementary references**

1. Stanciu, C. D. *et al*. All-optical magnetic recording with circularly polarized light. *Phys. Rev. Lett.* **99**, 047601 (2007).
2. Khorsand, A. R. *et al*. Role of magnetic circular dichroism in all-optical magnetic recording. *Phys. Rev. Lett.* **108**, 127205 (2012).
3. Ostler, T. A. *et al.* Ultrafast heating as a sufficient stimulus for magnetization reversal in a ferrimagnet. *Nat. Commun.* **3**, 666 (2012).
4. Vahaplar, K. *et al.* All-optical magnetization reversal by circularly polarized laser pulses: Experiment and multiscale modeling. *Phys. Rev. B* **85**, 104402 (2012).
5. Wilson, R. B. *et al.* Electron-phonon interaction during optically induced ultrafast magnetization dynamics of Au/GdFeCo bilayers. *arXiv:1609.00648* (2016).
6. Wilson, R. B., Apgar, B. A., Hsieh, W., Martin, L. W. & Cahill, D. G. Thermal conductance of strongly bonded metal-oxide interfaces. *Phys. Rev. B* **91**, 115414 (2015).
7. Ekici, O. *et al.* Thermal analysis of gold nanorods heated with femtosecond laser pulses. *J*. *Phys. D: Appl. Phys.* **41**, 185501 (2008).
8. Kryder, M. H. *et al.* Heat assisted magnetic recording. *Proc. IEEE* **96**, 1810 (2008).
9. Zhang, G., Hübner, W., Beaurepaire, E. & Bigot, J. Laser-Induced Ultrafast Demagnetization: Femtomagnetism, a New Frontier? *Topics Appl. Phys.* **83**, 245 (2002).

**Supplementary figures**


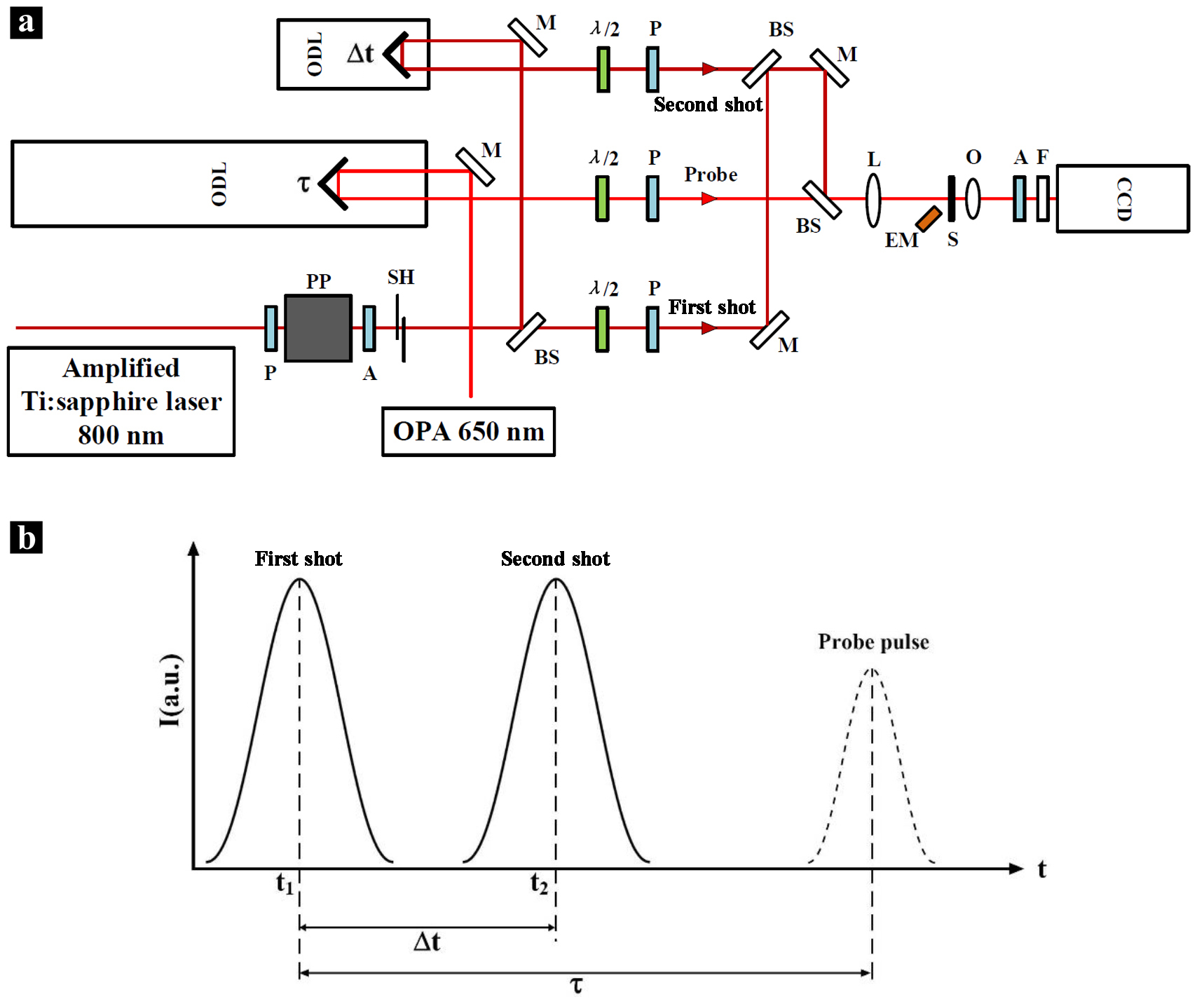


**Figure S1. (a)** More detailed schematic of the experimental setup (OPA: optical parametric amplifier, P: polariser, A: analyser, PP: pulse picker, SH: shutter, ODL: optical delay line, BS: beam splitter, M: mirror, *λ*/2: half-wave plate, L: focal lens, EM: electromagnet, S: sample, O: objective, F: colour filter, CCD: charge-coupled device). **(b)** Schematic of the time sequence of the time-resolved dynamics by dual-shot excitation. ∆t is the shot-to-shot separation between the first and the second shots and τ is the time delay between the probe pulse and the first pump pulse.


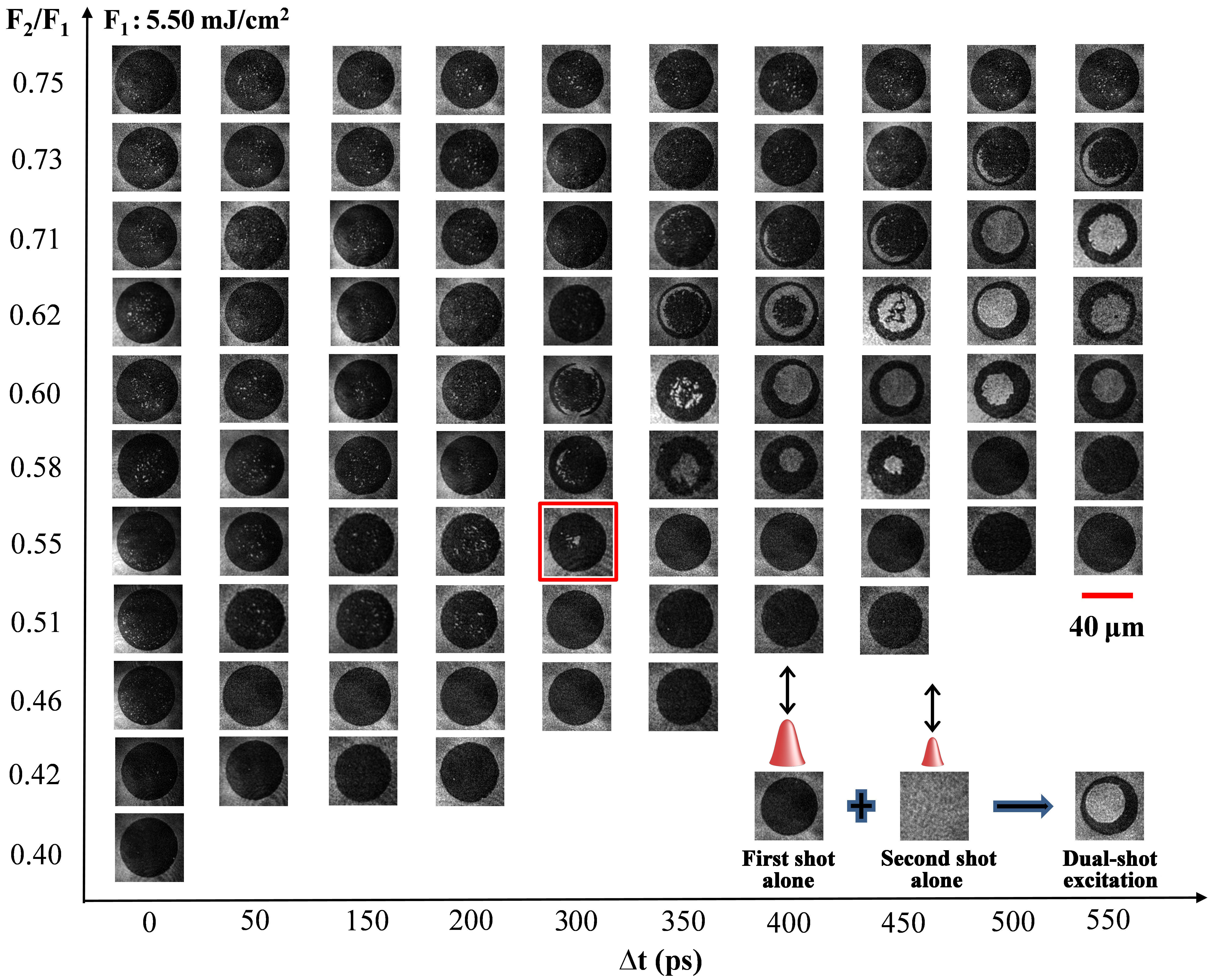


**Figure S2.** **The final state distributions of Gd_27_Fe_63.87_Co_9.13_ under dual-shot excitation versus ∆t and F_2_/F_1_ when the polarizations of the dual pulses are parallel.** The fluence of F_1_ is fixed at 5.50 mJ/cm^2^. The scale bar is 40 μm. It can be seen that the final state distributions under this condition are consistent with those shown in Fig. 2c in the main text. The red frame indicates the onset of restoring of the switched magnetization by the second shot after a minimal separation. The critical time delay ∆t_c_ is measured at around 300 ps as well. When ∆t is approaching or less than the pulse width, the two pumps may interfere within this time scale due to the parallel polarizations of the two pulses. Although no obvious difference is found between Fig. S2 and Fig. 2c within this time scale, orthogonal pump polarizations are selected to be shown in the main text to totally exclude the interference effect on the results.


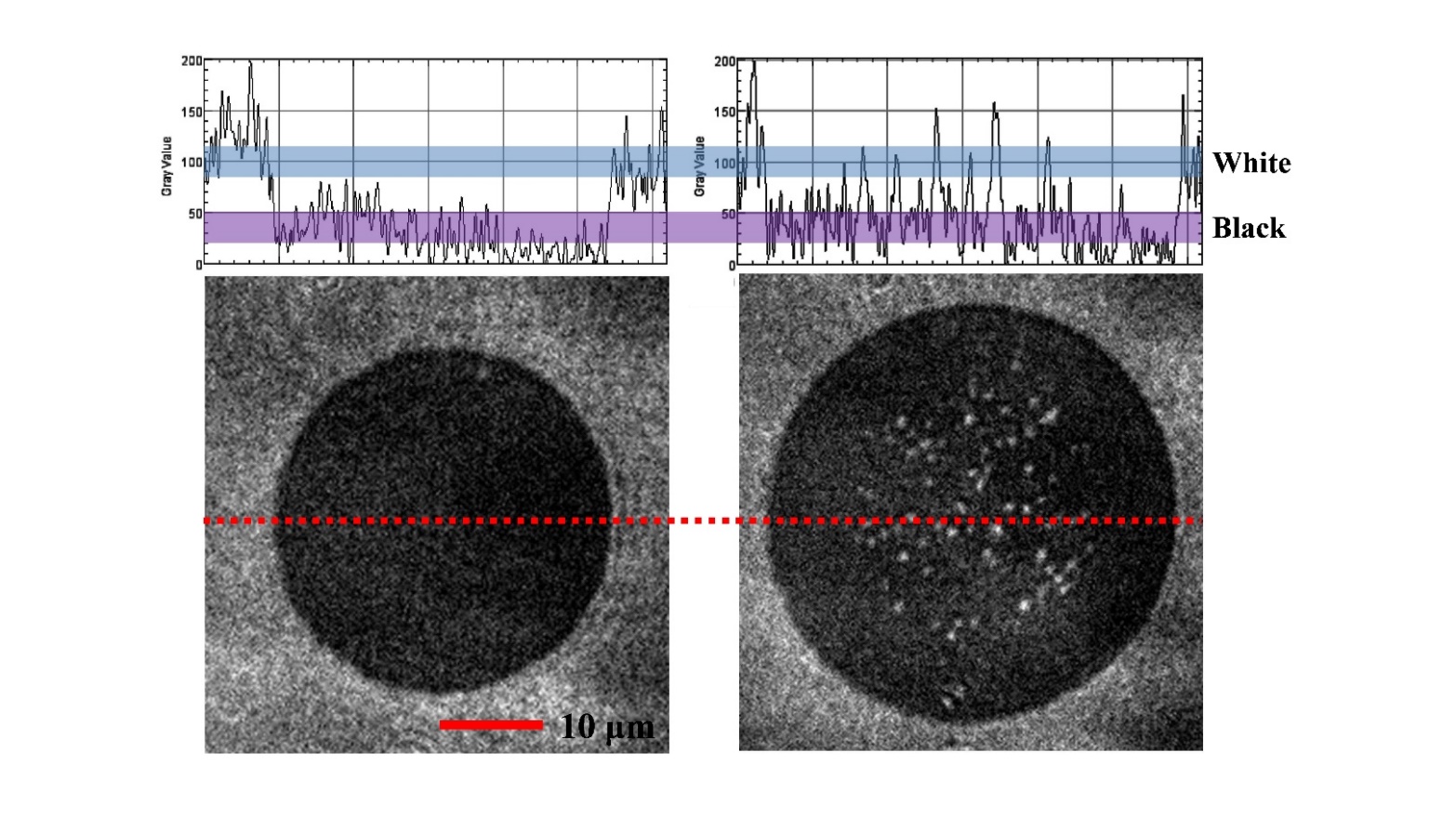


**Figure S3.** The cross-sections of the reversed magnetization and the multi-domain state along the dashed red line. The scale bar is 10 μm.


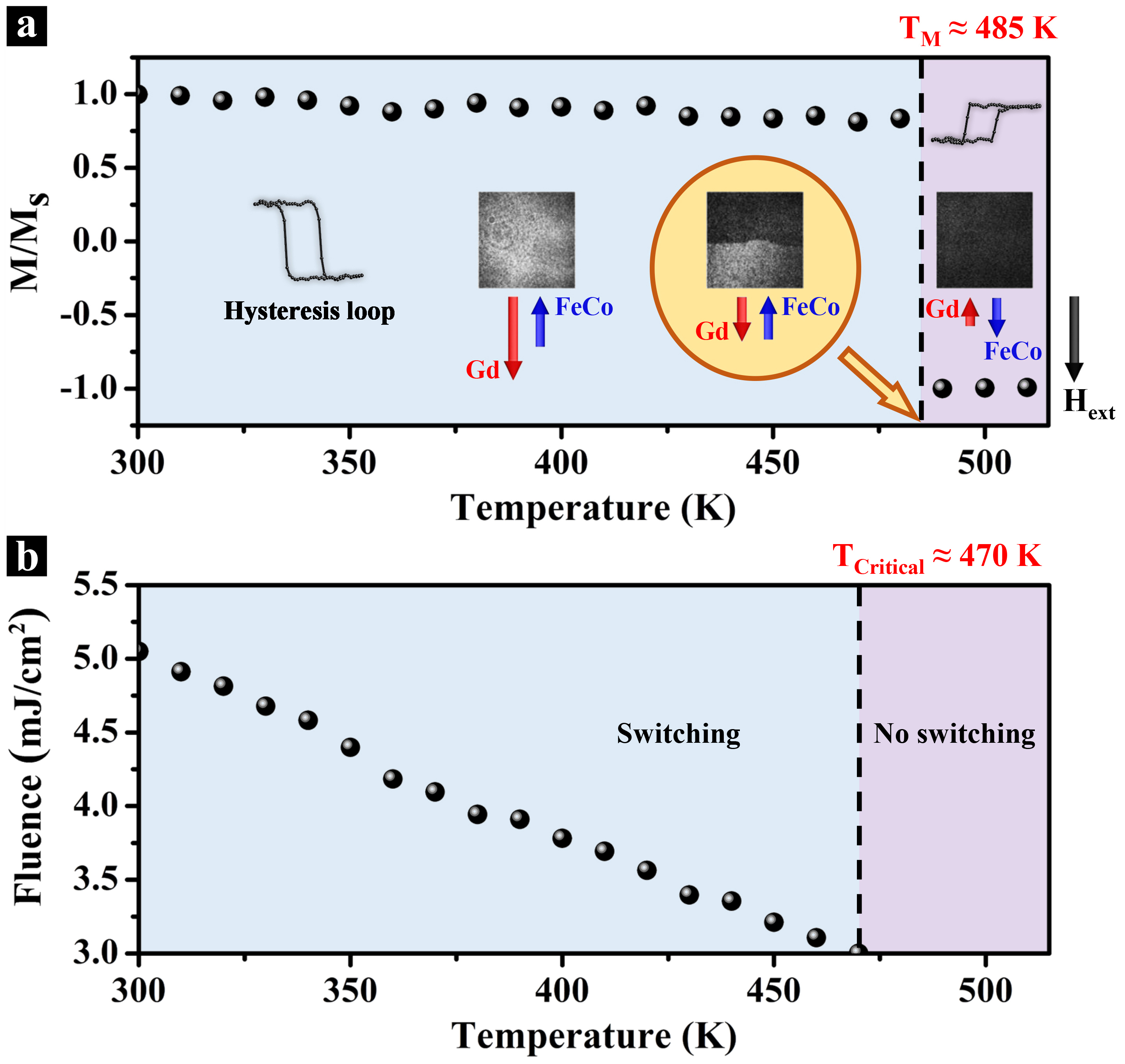


**Figure S4.** **(a)** The normalized magnetization of the FeCo sublattice at different temperatures with an out-of-plane external magnetic field of around 800 Oe. The insets show the measured hysteresis trajectories and the magnetization states of the FeCo sublattice during the heating process, where the red and blue arrows denote the magnetization of the Gd and FeCo sublattices, respectively. **(b)** The dependence of the minimum fluence for single-shot all-optical switching on the sample temperature.


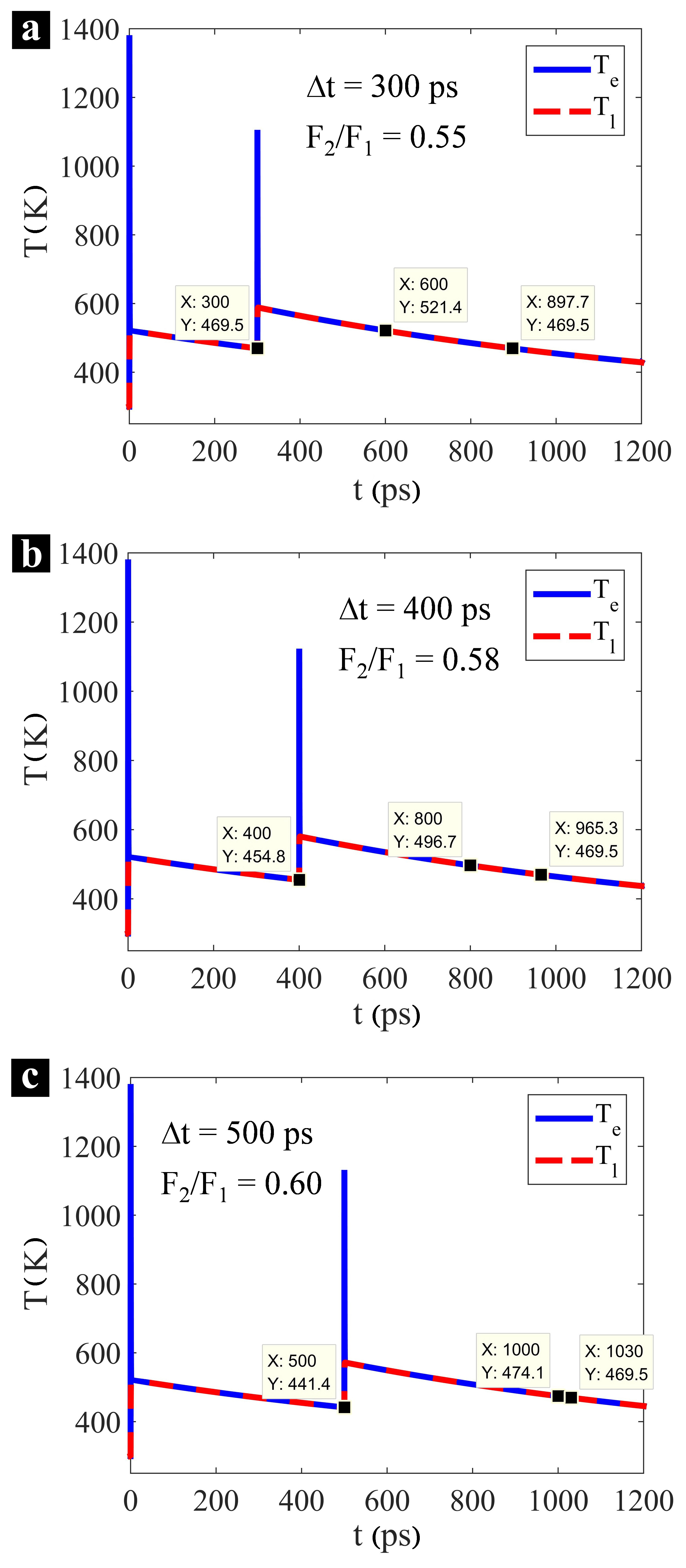


**Figure S5.** Time evolutions of the electron (solid blue line) and lattice (dashed red line) temperatures (T_e_ and T_l_) under dual-shot excitation with **(a)** ∆t = 300 ps, **(b)** ∆t = 400 ps, and **(c)** ∆t = 500 ps based on the two-temperature model. F_1_ = 5.50 mJ/cm^2^.


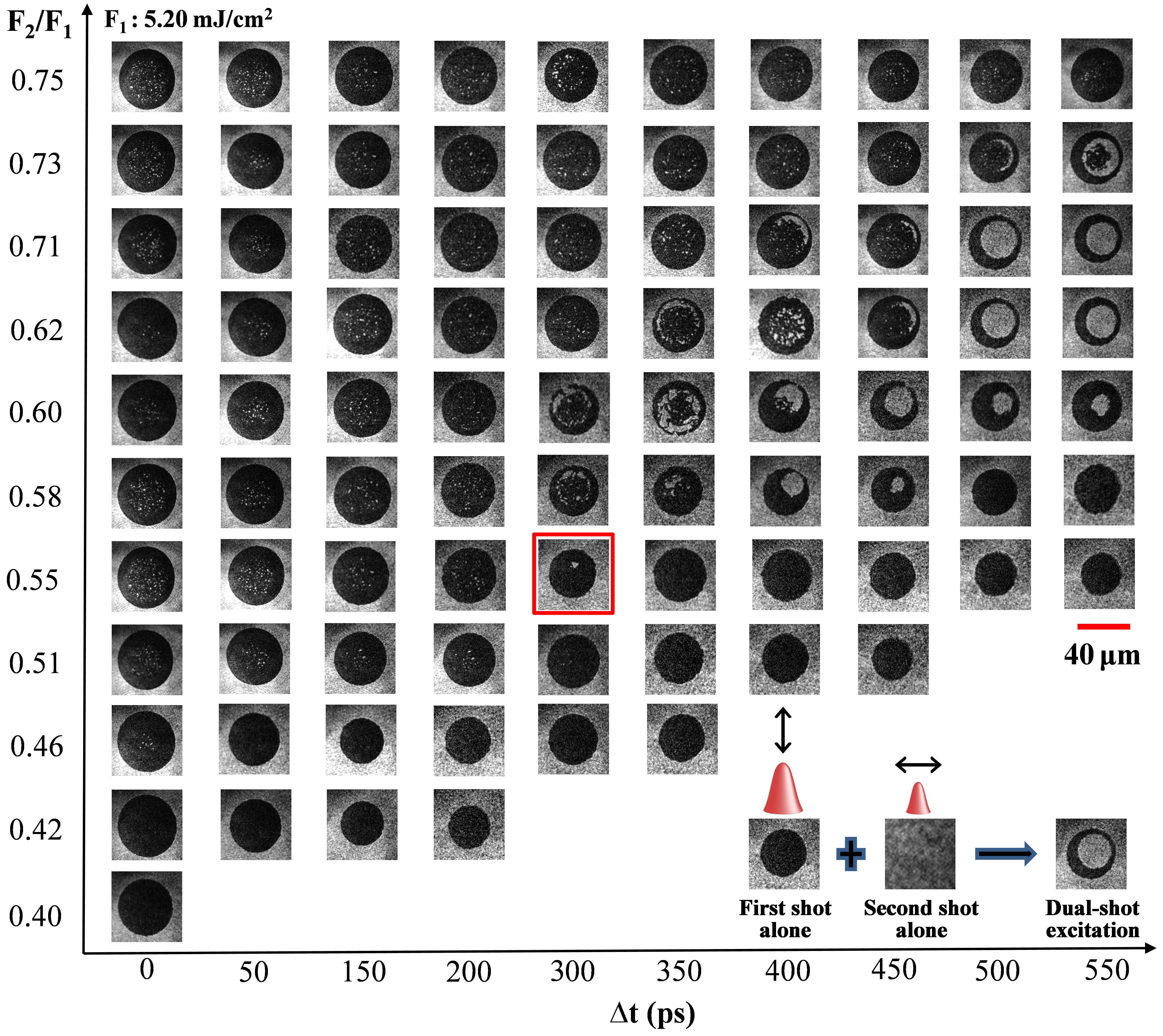


**Figure S6.** **The final state distributions of Gd_27_Fe_63.87_Co_9.13_ under dual-shot excitation versus ∆t and F_2_/F_1_ when the polarizations of the dual pulses are orthogonal.** The fluence of F_1_ is fixed at 5.20 mJ/cm^2^. The scale bar is 40 μm. The red frame indicates the onset of restoring of the switched magnetization by the second shot after a minimal separation. It can be seen that no obvious shift of ∆t_c_ is observed implying the critical time is independent of the fluence of the first shot.


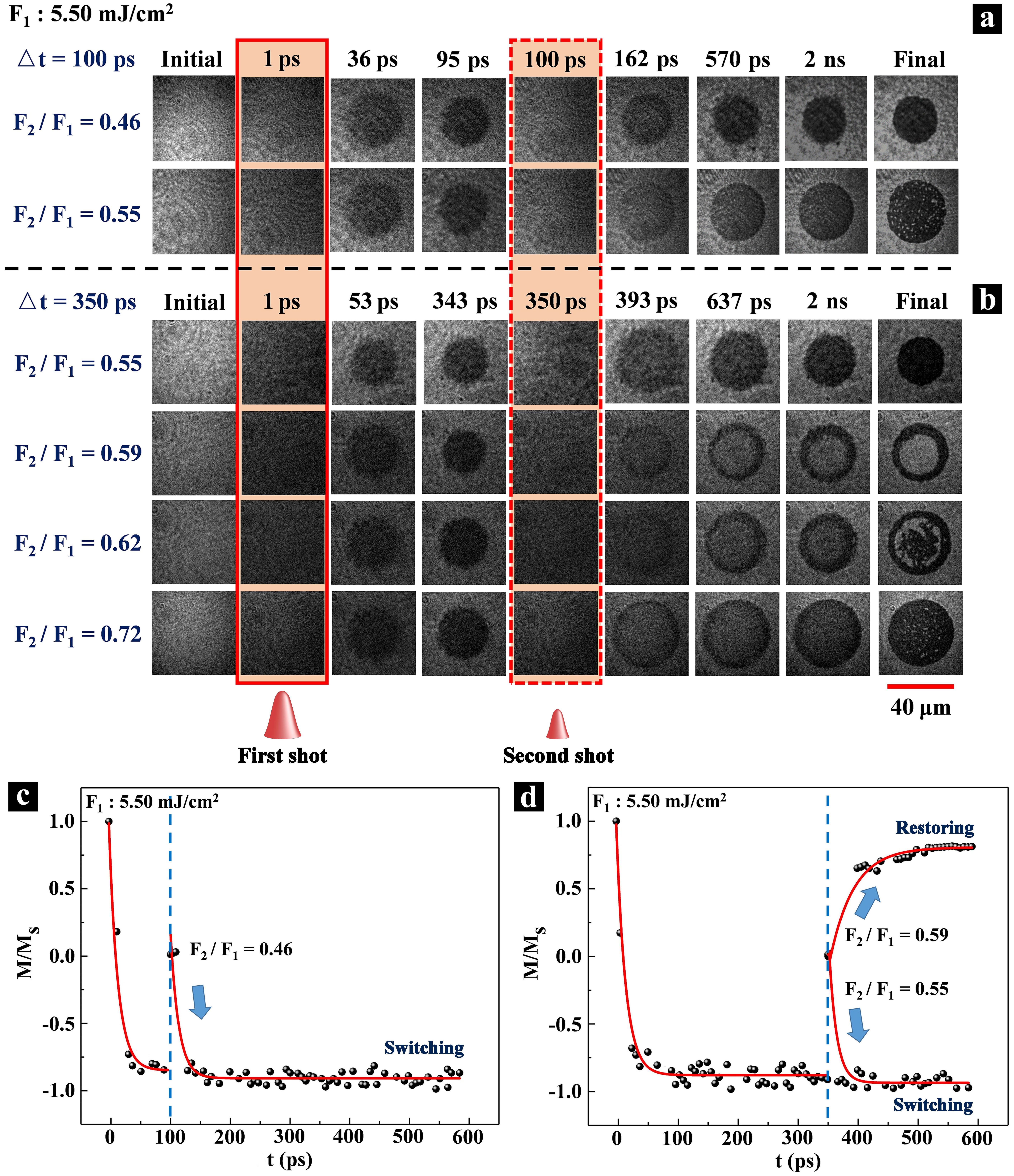


**Figure S7.** **(a)-(b)** The magnetization dynamics of Gd_27_Fe_63.87_Co_9.13_ under dual-shot excitation with other ∆t. The solid and dashed red frames indicate the images acquired at the arrivals of the first and second shots, respectively. The scale bar is 40 μm. **(c)-(d)** The normalized magnetization in the centre of the switched areas extracted from the images in **(a)-(b)**. ∆t in **(a)** and **(c)** and in **(b)** and **(d)** is 100 ps and 350 ps, respectively. Alike the magnetization evolution processes with ∆t being 200 ps or 450 ps, metastable sates of the magnetization are reached within 60 ps approximately after excitation of the second shot. Depending on the fluence ratios, the final magnetization returns to the switching state or turns to the restoring state.

**Supplementary movies**

Movie S1: The magnetization dynamics of Gd_27_Fe_63.87_Co_9.13_ under single-shot excitation when F_1_ = 5.50 mJ/cm^2^.

Movie S2: The magnetization dynamics of Gd_27_Fe_63.87_Co_9.13_ under dual-shot excitation when ∆t = 200 ps and F_2_/F_1_ = 0.46.

Movie S3: The magnetization dynamics of Gd_27_Fe_63.87_Co_9.13_ under dual-shot excitation when ∆t = 200 ps and F_2_/F_1_ = 0.55.

Movie S4: The magnetization dynamics of Gd_27_Fe_63.87_Co_9.13_ under dual-shot excitation when ∆t = 450 ps and F_2_/F_1_ = 0.55.

Movie S5: The magnetization dynamics of Gd_27_Fe_63.87_Co_9.13_ under dual-shot excitation when ∆t = 450 ps and F_2_/F_1_ = 0.58.

Movie S6: The magnetization dynamics of Gd_27_Fe_63.87_Co_9.13_ under dual-shot excitation when ∆t = 450 ps and F_2_/F_1_ = 0.67.

Movie S7: The magnetization dynamics of Gd_27_Fe_63.87_Co_9.13_ under dual-shot excitation when ∆t = 450 ps and F_2_/F_1_ = 0.72.
